# Tris(6-carb­oxy­pyridine-2-carboxyl­ato)terbium(III) 2.75-hydrate

**DOI:** 10.1107/S1600536811024135

**Published:** 2011-06-25

**Authors:** Soumaila Zebret, Céline Besnard, Josef Hamacek

**Affiliations:** a Department of Inorganic, Analytical, and Applied Chemistry, University of Geneva, 30, quai E. Ansermet, CH–1211 Geneva 4, Switzerland; b Laboratory of X-Ray Crystallography, University of Geneva, 24, quai E. Ansermet, CH–1211 Geneva 4, Switzerland

## Abstract

In the title compound, [Tb(C_7_H_4_NO_4_)_3_]·2.75H_2_O, the Tb^3+^ atom is coordinated by three tridentate 6-carb­oxy­pyridine-2-carboxyl­ate ligands and lies on a crystallographic threefold rotation axis. The coordination polyhedron around Tb^III^ adopts a distorted tricapped trigonal–prismatic geometry. Disordered water mol­ecules with partial occupancy are also present in the crystal, one of which is associated with each of the carboxyl­ate O atoms of the complex unit.

## Related literature

For details of the synthesis, see: Zebret *et al.* (2009 ▶). For related structures, see: D’Aléo, *et al.* (2007 ▶, 2008 ▶); Borthwick (1980 ▶); Albertsson (1970 ▶); Hamacek *et al.* (2009 ▶). For isotypic structures, see: Brayshaw *et al.* (2005 ▶); Chen *et al.* (2002 ▶); Iwamura *et al.* (2007 ▶); Lunstroot *et al.* (2009 ▶); Pompidor *et al.* (2008 ▶); Shengzhi *et al.* (1989 ▶); Van Meervelt *et al.* (1997 ▶). For the Squeeze/bypass procedure, see: van der Sluis & Spek (1990 ▶). For a description of the Cambridge Structural Database, see: Allen (2002) ▶.
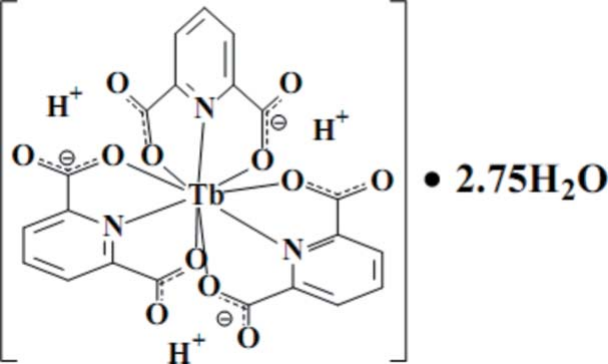


## Experimental

### 

#### Crystal data

[Tb(C_7_H_4_NO_4_)_3_]·2.75H_2_O
*M* *_r_* = 706.79
Trigonal, 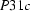

*a* = 13.0115 (15) Å
*c* = 9.4142 (13) Å
*V* = 1380.3 (5) Å^3^
*Z* = 2
Mo *K*α radiation
μ = 2.63 mm^−1^
*T* = 200 K
0.15 × 0.10 × 0.05 mm

#### Data collection

Stoe IPDS diffractometer
Absorption correction: Gaussian (Busing & Levy, 1957 ▶) *T*_min_ = 0.72, *T*_max_ = 0.88
3830 measured reflections
1569 independent reflections
1464 reflections with *I* > 2.0σ(*I*)
*R*_int_ = 0.035

#### Refinement

*R*[*F* ^2^ > 2σ(*F* ^2^)] = 0.037
*wR*(*F* ^2^) = 0.082
*S* = 1.00
1566 reflections
122 parameters
1 restraint
H-atom parameters constrained
Δρ_max_ = 0.57 e Å^−3^
Δρ_min_ = −1.01 e Å^−3^
Absolute structure: Flack (1983 ▶), 679 Friedel pairs
Flack parameter: −0.05 (2)

### 

Data collection: *IPDS* (Stoe & Cie, 1996 ▶); cell refinement: *IPDS*; data reduction: *X-RED* (Stoe & Cie 1996 ▶); program(s) used to solve structure: *SIR92* (Altomare *et al.*, 1994 ▶); program(s) used to refine structure: *CRYSTALS* (Betteridge *et al.*, 2003 ▶); molecular graphics: *CAMERON* (Watkin *et al.*, 1996) ▶; software used to prepare material for publication: *CRYSTALS*.

## Supplementary Material

Crystal structure: contains datablock(s) global, I. DOI: 10.1107/S1600536811024135/zs2104sup1.cif

Structure factors: contains datablock(s) I. DOI: 10.1107/S1600536811024135/zs2104Isup2.hkl

Additional supplementary materials:  crystallographic information; 3D view; checkCIF report

## Figures and Tables

**Table 1:** Selected bond lengths (Å)

Tb1—O2	2.435 (6)
Tb1—N6	2.545 (6)
Tb1—O9	2.436 (6)

Symmetry codes: (i) 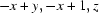; (ii) 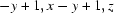.

**Fig. 1.**
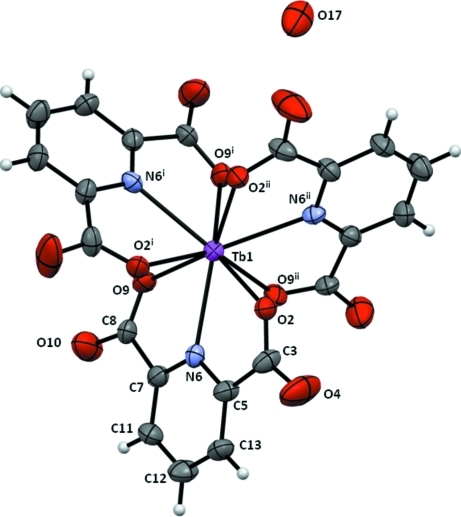
ORTEP view of the terbium tris(dipicolinate) complex (along the c axis) showing the threefold symmetry, the atom-numbering and the displacement ellipsoids with 50% probability. For symmetry codes: (i) -x + y, -x + 1, z; (ii) -y + 1, x-y + 1, z.

**Fig. 2.**
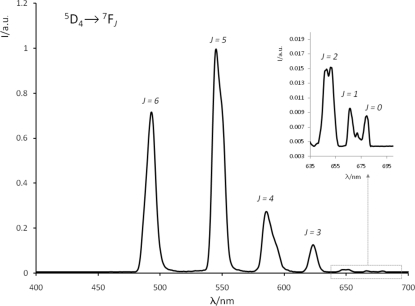
Phosphorescence spectrum of the title compound (solid state, RT).

**Fig. 3.**
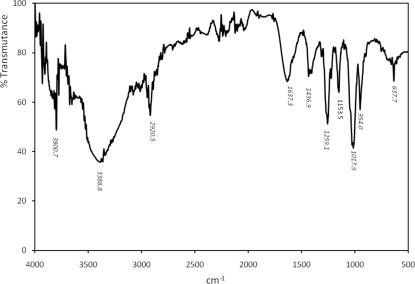
IR spectrum of the title compound (solid state, RT).

**Fig. 4.**
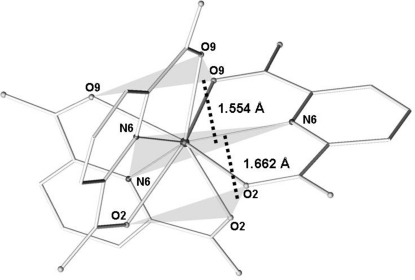
Schematic representation of three facial planes containing donor atoms in title compound.

**Fig. 5.**
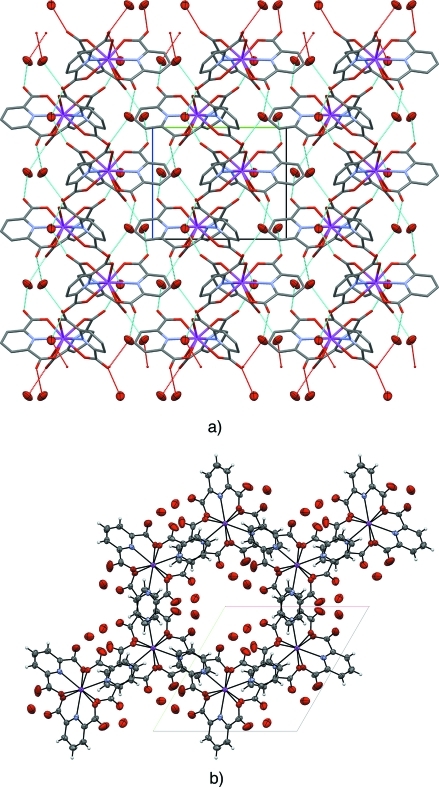
Crystal packing of the title compound showing the unit-cell contents and the disordered partial water molecules in the channels (a) viewed along the c axis and (b) viewed along the a axis.

## supplementary crystallographic information

### Comment

Since the first structural investigation of tris(dipicolinato)ytterbium complex (Albertsson, 1970), a number of different lanthanide complexes with *dipic* (= hydrogen 2,6-pyridinedicarboxylate) have been reported. Their brief overview can be found, e.g. in the report of the Hamacek group (Hamacek *et al.*, 2009). Recently, we have reported on the self-assembly of a trinuclear luminescent europium complex with bis(6-methoxycarbonyl-2-carbonylpyridine)amine (*L*) (Zebret *et al.*, 2009). In an attempt to synthesize the analogous terbium(III) compound with *L* using the same procedure, small transparent crystals were isolated from the resulting DMSO solution. However, these X-ray quality crystals had a different shape than expected (cubic crystals for Eu_3_*L*_3_). Structural studies reveal the formation of a partially hydrated tris(dipicolinato^-1^)terbium complex, the title compound [Tb(*dipic*)_3_] . 2.75H_2_O (I) (Fig. 1). Accordingly, the presence of dipicolinate anions is explained by the complete hydrolysis of both ester and amide functions of the ligand *L*. In addition to crystallography, the obtained crystalline material was analysed using spectroscopic methods. Fig. 2 shows the typical emission spectrum of [Tb(*dipic*)_3_]^3-^ with characteristic ^5^D_4_ -> ^7^F_j_ transitions (D'Aléo *et al.*, 2007, 2008). The luminescent lifetime at room temperature was found to be 1.45 ms, which is a somewhat lower value compared to the D'Aleo's value (2.02 ms) probably due to additional quenching of surrounding water molecules. The IR spectrum on Fig. 3 shows stretching O—H vibrations at about 3400 cm^-1^ and water bending vibrations at 1637 cm^-1^.

From the bond lengths, the valence of the Tb atom was calculated to be 3.15. Taking into account the oxidation state of the Tb atom and in the absence of any other charged species in the crystals, the ligand has to be partially protonated. However, the quality of the data does not allow the localion of the position of this extra hydrogen on each of the ligands. Apart from the complex, additional partial water molecules are present in the crystal, one of which (O17) is associated with the carboxyl oxygens of the ligand  (details of the refinement are given elsewhere).

In the complex, the Tb^III^ cation is nine-coordinated by three monoanionic *dipic*^1-^ ligands and lies on the threefold rotation axis. The Tb atom lies 0.081 Å  from the plane defined by the three nitrogen donors, so that these four atoms are nearly coplanar (Fig. 4). The two planes defined by the three O2 and three O9 donors, respectively, form a discrete tricapped trigonal prism with the distance to the central terbium atom equal to 1.581 Å and 1.635 Å, respectively. The asymmetric unit comprises one dipicolinate ligand and 1/3 of a Tb atom and the partial water O17 (S.O.F = 0.50). The unit cell consists of two molecules of [Tb(*dipic*)_3_] . 2.75H_2_O with two different configurations (Δ and Λ) for the complex unit; the crystal is thus a racemate. The overall structure of the Tb^III^ complex does not significantly differ from those previously reported for other lanthanides. A search of the Cambridge Structural Database [CSD, Version 5.30 of September 2009 (Allen, 2002)] restricted only to tris(dipicolinato)terbium complexes gives eight crystal structures already reported in the literature, details of which are summarized in Table 2. As it can be seen, the Tb—O distances are not completely symmetrical within the complex ranging from 2.38 to 2.45 Å. Similar results are found for complex (I) (Table 1) with the Tb—O2 and Tb—O9 distances equal to 2.434 (6) Å and 2.437 (6) Å, respectively. The Tb—N6 distance is equal to 2.544 (6) Å, apparently the longest distance for all reported structures.

Concerning the crystal packing, the [Tb(*dipic*)_3_] units are arranged in the plane around disordered partial water molecules, occupying the available spaces, which resemble channels (Fig. 5a). The underlayer is shifted along the *b* axis in order to optimize hydrogen bonding interactions (Fig. 5b). A comparison of structural data in Table 2 shows that the choice of the counter-ion [absent in the case of (I)] has a significant influence on the final crystal packing (seven space groups for nine tris(dipicolinato)terbium complexes), and also on the coordinate bonds within the complex (various Tb—N and Tb—O distances).

### Experimental

To a solution of 60.8 mg (0.18 mmol) of the pyridine-containing ligand *L* and 152.5 mg (0.18 mmol) of Tb(Otf)_3_ . 13.7H_2_O in 5 ml DMF was added NaH (26 mg 3.5 eq). The mixture was stirred for three hours under a nitrogen atmosphere, filtered and then evaporated to dryness. The residue was dissolved in DMSO, filtered and water was allowed to diffuse into this solution. The IR spectrum of the isolated solid was measured at room tempeature with a Perkin-Elmer Spectrum 1 (equipped with a Specac Golden Gate ATR accessory). The phosphorescence spectrum was obtained under the same conditions with a Perkin-Elmer Lambda 900 (λ_exc_ = 273 nm).

### Refinement

In the absence of any other element that could satisfactorily fit the solvent peaks in the Fourier difference map, the solvent density was attributed to partially occupied water molecules. One of them was included in the model. Its occupancy was refined to 0.45 with *U*_iso_ fixed to 0.05 and then fixed to 0.5 while refining the anisotropic displacement parameters. The other possible water molecules that were seen in the solvent density had low occupancies and large anisotropic displacement parameters. The Squeeze/bypass procedure (Sluis *et al.*, 1990) was therefore used to take care of the extra electron density in the channel. 26 Electrons were found in a void of 202 Å^3^. This is compatible with the presence of 2.5 extra water molecules per unit-cell that were added to the formula. Concerning the charge of the complex, a model with a protonated COOH ligand making a neutral complex is the most probable since infrared measurement and the synthesis conditions do not suggest the presence of an oxonium ion. Although some density is found in the final difference Fourier map around O2 and O4, the geometry of both COO^-^ group and especially their symmetry and bond length do not allow to conclude unambiguously on the position of the extra hydrogen in the ligand. As a consequence, it was not included in the model. Short O17—O contacts [2.84 (2) Å to O10 and 2.85 (2) Å to O4] indicate possible hydrogen bonds between O17 and O4 and O10. O4 and O10 are potential candidates to accommodate the extra proton on the ligand and can act as donors for these hydrogen bonds. If not protonated, they would suit as acceptors for hydrogen bonds involving the hydrogen of the water molecule containing O17. The extra water molecules present in the structure that are not included in the model may also participate to the hydrogen-bonding network.

### Crystal data

**Table:**

[Tb(C_7_H_4_NO_4_)_3_]·2.75H_2_O	*D*_x_ = 1.701 Mg m^−^^3^
*M**_r_* = 706.79	Mo *K*α radiation, λ = 0.71073 Å
Trigonal, *P*31*c*	Cell parameters from 4000 reflections
Hall symbol: P 3 -2c	θ = 2.8–32.1°
*a* = 13.0115 (15) Å	µ = 2.63 mm^−^^1^
*c* = 9.4142 (13) Å	*T* = 200 K
*V* = 1380.3 (5) Å^3^	Prism, colourless
*Z* = 2	0.15 × 0.10 × 0.05 mm
*F*(000) = 694.85	

### Data collection

**Table:**

Stoe IPDS diffractometer	1464 reflections with *I* > 2.0σ(*I*)
graphite	*R*_int_ = 0.035
ω scans	θ_max_ = 25.8°, θ_min_ = 2.8°
Absorption correction: gaussian (Busing & Levy, 1957)	*h* = −13→15
*T*_min_ = 0.72, *T*_max_ = 0.88	*k* = −12→15
3830 measured reflections	*l* = −10→11
1569 independent reflections	

### Refinement

**Table:**

Refinement on *F*^2^	Hydrogen site location: inferred from neighbouring sites
Least-squares matrix: full	H-atom parameters constrained
*R*[*F*^2^ > 2σ(*F*^2^)] = 0.037	*w* = 1/[σ^2^(*F*_o_^2^) + (0.03*P*)^2^ + 8.38*P*] where *P* = (max(*F*_o_^2^,0) + 2*F*_c_^2^)/3
*wR*(*F*^2^) = 0.082	(Δ/σ)_max_ = 0.009
*S* = 1.00	Δρ_max_ = 0.57 e Å^−^^3^
1566 reflections	Δρ_min_ = −1.01 e Å^−^^3^
122 parameters	Absolute structure: Flack (1983), 679 Friedel pairs: the crystal is achiral.
1 restraint	Flack parameter: −0.05 (2)
Primary atom site location: structure-invariant direct methods	

### Special details

**Table:**

Experimental. The crystal was placed in the cold stream of an Oxford Cryosystems open-flow nitrogen cryostat (Cosier & Glazer, 1986) with a nominal stability of 0.1 K.Cosier, J. & Glazer, A.*M*., 1986. J. Appl. Cryst. 105 107.

### Fractional atomic coordinates and isotropic or equivalent isotropic displacement parameters (Å^2^)

**Table:**

	*x*	*y*	*z*	*U*_iso_*/*U*_eq_	Occ. (<1)
Tb1	0.3333	0.6667	0.38705 (17)	0.0281	
O2	0.4896 (5)	0.7887 (6)	0.2190 (6)	0.0368	
C3	0.5468 (8)	0.9002 (8)	0.2214 (10)	0.0390	
O4	0.6394 (7)	0.9631 (7)	0.1429 (9)	0.0940	
C5	0.5045 (7)	0.9629 (7)	0.3171 (8)	0.0342	
N6	0.4104 (5)	0.8890 (6)	0.3959 (7)	0.0284	
C7	0.3641 (7)	0.9320 (8)	0.4896 (9)	0.0310	
C8	0.2606 (7)	0.8377 (8)	0.5728 (8)	0.0345	
O9	0.2404 (5)	0.7332 (5)	0.5607 (6)	0.0366	
O10	0.2028 (7)	0.8686 (6)	0.6551 (8)	0.0659	
C11	0.4116 (7)	1.0542 (7)	0.5101 (9)	0.0392	
C12	0.5099 (9)	1.1317 (8)	0.4296 (10)	0.0450	
C13	0.5560 (8)	1.0869 (8)	0.3313 (9)	0.0412	
H111	0.3784	1.0822	0.5748	0.0471*	
H121	0.5442	1.2128	0.4413	0.0541*	
H131	0.6209	1.1381	0.2763	0.0490*	
O17	0.2402 (15)	0.1736 (16)	0.4073 (16)	0.0795	0.5000

### Atomic displacement parameters (Å^2^)

**Table:**

	*U*^11^	*U*^22^	*U*^33^	*U*^12^	*U*^13^	*U*^23^
Tb1	0.02359 (17)	0.02359 (17)	0.0372 (3)	0.01180 (8)	0.0000	0.0000
O2	0.032 (3)	0.034 (4)	0.045 (3)	0.017 (3)	0.008 (2)	−0.002 (3)
C3	0.031 (5)	0.032 (5)	0.040 (5)	0.006 (4)	0.009 (4)	−0.002 (4)
O4	0.066 (6)	0.060 (5)	0.119 (7)	0.003 (4)	0.049 (5)	−0.016 (5)
C5	0.032 (4)	0.028 (4)	0.035 (4)	0.009 (3)	0.002 (3)	0.001 (3)
N6	0.025 (3)	0.032 (3)	0.028 (3)	0.015 (3)	0.005 (3)	0.000 (3)
C7	0.027 (5)	0.028 (4)	0.037 (5)	0.012 (4)	−0.001 (3)	0.003 (3)
C8	0.025 (4)	0.037 (5)	0.043 (5)	0.017 (4)	0.004 (3)	0.000 (3)
O9	0.031 (3)	0.026 (3)	0.048 (3)	0.011 (3)	0.013 (3)	0.007 (3)
O10	0.064 (5)	0.053 (4)	0.087 (5)	0.034 (4)	0.035 (4)	0.011 (4)
C11	0.041 (5)	0.030 (4)	0.048 (5)	0.019 (4)	0.001 (3)	−0.005 (3)
C12	0.047 (5)	0.026 (5)	0.052 (6)	0.011 (4)	0.003 (4)	−0.009 (4)
C13	0.034 (5)	0.024 (4)	0.055 (5)	0.007 (4)	0.008 (4)	0.004 (4)
O17	0.071 (11)	0.098 (13)	0.071 (10)	0.043 (10)	−0.007 (8)	−0.044 (9)

### Geometric parameters (Å, °)

**Table:**

Tb1—N6^i^	2.545 (6)	C5—N6	1.340 (9)
Tb1—N6^ii^	2.545 (6)	C5—C13	1.410 (12)
Tb1—O9^ii^	2.436 (6)	N6—C7	1.338 (11)
Tb1—O9^i^	2.436 (6)	C7—C8	1.510 (11)
Tb1—O2^i^	2.435 (6)	C7—C11	1.402 (11)
Tb1—O2^ii^	2.435 (6)	C8—O9	1.253 (10)
Tb1—O2	2.435 (6)	C8—O10	1.277 (10)
Tb1—N6	2.545 (6)	C11—C12	1.392 (12)
Tb1—O9	2.436 (6)	C11—H111	0.921
O2—C3	1.257 (11)	C12—C13	1.381 (12)
C3—O4	1.297 (11)	C12—H121	0.924
C3—C5	1.494 (12)	C13—H131	0.929
			
N6^i^—Tb1—N6^ii^	119.893 (19)	O2^ii^—Tb1—N6	74.7 (2)
N6^i^—Tb1—O9^ii^	70.1 (2)	O2—Tb1—N6	63.52 (19)
N6^ii^—Tb1—O9^ii^	63.73 (19)	O2^ii^—Tb1—O9	83.45 (18)
N6^i^—Tb1—O9^i^	63.73 (19)	O2—Tb1—O9	127.2 (2)
N6^ii^—Tb1—O9^i^	135.8 (2)	N6—Tb1—O9	63.73 (19)
O9^ii^—Tb1—O9^i^	79.9 (2)	Tb1—O2—C3	124.1 (5)
N6^i^—Tb1—O2^i^	63.52 (19)	O2—C3—O4	123.1 (8)
N6^ii^—Tb1—O2^i^	74.7 (2)	O2—C3—C5	118.3 (7)
O9^ii^—Tb1—O2^i^	83.45 (18)	O4—C3—C5	118.6 (8)
O9^i^—Tb1—O2^i^	127.2 (2)	C3—C5—N6	113.3 (7)
N6^i^—Tb1—O2^ii^	140.7 (2)	C3—C5—C13	126.0 (7)
N6^ii^—Tb1—O2^ii^	63.52 (19)	N6—C5—C13	120.7 (8)
O9^ii^—Tb1—O2^ii^	127.2 (2)	Tb1—N6—C5	119.8 (5)
O9^i^—Tb1—O2^ii^	144.8 (2)	Tb1—N6—C7	119.5 (5)
O2^i^—Tb1—O2^ii^	82.3 (2)	C5—N6—C7	120.3 (7)
N6^i^—Tb1—O2	74.7 (2)	N6—C7—C8	114.0 (7)
N6^ii^—Tb1—O2	140.7 (2)	N6—C7—C11	122.0 (8)
O9^ii^—Tb1—O2	144.8 (2)	C8—C7—C11	123.9 (8)
O9^i^—Tb1—O2	83.45 (18)	C7—C8—O9	116.9 (7)
O2^i^—Tb1—O2	82.3 (2)	C7—C8—O10	119.0 (8)
N6^i^—Tb1—N6	119.893 (19)	O9—C8—O10	124.0 (7)
N6^ii^—Tb1—N6	119.893 (18)	Tb1—O9—C8	125.0 (5)
O9^ii^—Tb1—N6	135.8 (2)	C7—C11—C12	118.1 (8)
O9^i^—Tb1—N6	70.1 (2)	C7—C11—H111	120.8
O2^i^—Tb1—N6	140.7 (2)	C12—C11—H111	121.0
N6^i^—Tb1—O9	135.8 (2)	C11—C12—C13	119.6 (8)
N6^ii^—Tb1—O9	70.1 (2)	C11—C12—H121	120.4
O9^ii^—Tb1—O9	79.9 (2)	C13—C12—H121	119.9
O9^i^—Tb1—O9	79.9 (2)	C5—C13—C12	119.2 (7)
O2^i^—Tb1—O9	144.8 (2)	C5—C13—H131	120.7
O2^ii^—Tb1—O2	82.3 (2)	C12—C13—H131	120.1

Symmetry codes: (i) −*x*+*y*, −*x*+1, *z*; (ii) −*y*+1, *x*−*y*+1, *z*.

### Hydrogen-bond geometry (Å, °)

**Table:**

*D*—H···*A*	*D*—H	H···*A*	*D*···*A*	*D*—H···*A*
C13—H131···O9^iii^	0.93	2.47	3.345 (13)	158

Symmetry codes: (iii) *y*, *x*+1, *z*−1/2.

### Table 2 Overview of crystal structures containing the tris(dipicolinato)terbium(III) complex unit.

**Table:**

*Space group*	*Counter-ion*	*d*(Tb-N)	*d*(Tb-O1)	*d*(Tb-O2)
				
P-1	[Co(NH_3_)_6_]^3+^*^a^*	2.509	2.416	2.446
		2.495	2.428	2.428
		2.492	2.408	2.419
P-1	[N(CH_2_CH_2_NH_3_)]^3+^*^b^*	2.492	2.398	2.431
		2.509	2.393	2.448
		2.499	2.428	2.435
P-1	[(NH_2_)_2_CNHCH_2_CH_3_)]^+^*^c^*	2.500	2.403	2.410
		2.491	2.402	2.429
		2.544	2.383	2.453
P2_1_	[Co(NH_2_CH_2_CH_2_NH_2_)_3_]^3+^*^d^*	2.533	2.412	2.418
		2.505	2.413	2.440
		2.508	2.417	2.431
		2.505	2.418	2.432
		2.512	2.432	2.441
		2.486	2.402	2.423
P2_1_/c	Na^+^*^e^*	2.504	2.406	2.415
		2.509	2.424	2.429
		2.505	2.410	2.439
C2/c	[Co(NH_2_CH_2_CH_2_NH_2_)_3_]^3+^*^d^*	2.486	2.400	2.423
		2.509	2.419	2.429
		2.518	2.407	2.416
P3_1_c	H^+^*^f^*	2.548	2.426	2.436
		2.547	2.426	2.435
		2.546	2.425	2.435
R-3c	[(CH_3_)_3_NCH_2_CH_2_OH)]^+^*^g^*	2.497	2.401	2.405
		2.496	2.401	2.404
		2.497	2.402	2.405
P-62c	Na^+^, ClO_4_^-^*^h^*	2.489	2.400	2.400

Notes: (*a*) Brayshaw *et al.* (2005); (*b*) Chen *et al.* (2002); (c) Pompidor *et al.* (2008); (*d*) Iwamura *et al.* (2007); (*e*) Shengzhi *et al.* (1989); (*f*) this work; (*g*) Lunstroot *et al.* (2009); (*h*) Van Meervelt *et al.* (1997).
